# The SGK1/ENaC axis in renal injury during salt-sensitive hypertension: from sodium sensing to inflammation

**DOI:** 10.3389/fimmu.2026.1839838

**Published:** 2026-06-19

**Authors:** Yan Wang, Meixiang Wu, Jinlan Ma, Ruixin Liu, Delong Duo

**Affiliations:** 1 Qinghai University Affiliated Hospital, Xining, China; 2 Qinghai Hospital, Xining, China. The First Teaching Hospital of Tianjin University of Traditional Chinese Medicine, Tianjin, China; 3 School of Medicine, Qinghai University, Xining, China; 4 Qinghai Provincial People’s Hospital, Xining, China

**Keywords:** antigen-presenting cells, epithelial sodium channel, inflammation, renal injury, salt-sensitive hypertension, SGK1, sodium sensing

## Abstract

Salt-sensitive hypertension (SSHT) is commonly framed as a disorder of renal sodium transport and vascular dysfunction. Yet increasing evidence indicates that immune cells also detect sodium excess and convert it into inflammatory signaling. In this article, we argue that the serum and glucocorticoid-regulated kinase 1 (SGK1)/epithelial sodium channel (ENaC) immune axis provides a useful framework for linking salt exposure to renal injury. In antigen-presenting cells (APCs), high salt activates SGK1, increases ENaC expression and sodium entry, and promotes oxidative stress, isolevuglandin formation, inflammasome activation, and cytokine release. In T-lymphocytes (T-cells), SGK1 supports pathogenic effector responses and contributes to hypertensive end-organ inflammation. Together, these observations suggest that immune sodium sensing is not merely a consequence of elevated blood pressure but an active process that sustains renal inflammation and maladaptive remodeling. We further propose that this pathway may help explain how immune–tubular crosstalk amplifies kidney injury in salt-sensitive hypertension. Defining when and where SGK1/ENaC signaling operates across renal immune cell subsets may open new opportunities for targeted therapy.

## 1 Introduction

Salt-sensitive hypertension (SSHT) is a clinical phenotype of hypertension in which blood pressure rises excessively in response to increased dietary salt intake or salt loading (1). In many related studies, SSHT is commonly defined as a rise in blood pressure (BP) of about 10% or more when a person changes from a low-salt diet to a high-salt diet (2). In animal studies, high-salt diets typically contain 4%-8% NaCl, whereas in cell culture studies, high-salt conditions are usually modeled using 145–160 mM NaCl, concentrations that are closer to pathological levels observed *in vivo*. In contrast, 190 mM NaCl represents a hyperphysiological and hyperosmolar condition and was therefore excluded (3). And approximately 50% of hypertensive patients and 25% of individuals with normal blood pressure exhibit salt sensitivity to blood pressure (SSBP) (4). These numbers suggest that salt sensitivity is not a marginal phenomenon, but a common and clinically relevant trait that may substantially increase the burden of cardiovascular and renal disease.

Salt-sensitive hypertension (SSHT) has long been attributed to impaired renal sodium excretion and abnormal vascular adaptation (5, 6). In this traditional view, the kidney occupies a central position because reduced sodium excretion promotes sodium retention, extracellular volume expansion, and a sustained rise in blood pressure (7, 8). Emerging evidence indicates that immune cells directly sense extracellular sodium elevation and initiate inflammatory signaling, independent of blood pressure changes.

In recent years, growing evidence has shown that immune cells have the ability to sense changes in the sodium environment (9). In antigen-presenting cells (APCs), sodium entry through amiloride-sensitive epithelial sodium channels (ENaC) can trigger oxidative stress, isolevuglandin adduct formation, inflammasome activation, and cytokine release (9–11). This response is further strengthened by serum and glucocorticoid-regulated kinase 1 (SGK1), which promotes ENaC activity and contributes to renal inflammation in experimental SSHT (12). In parallel, SGK1 in T- lymphocytes (T-cell) exists pathogenic effector responses and has been linked to hypertensive end-organ damage (12, 13).

This article will explore the following five key issues:

1. Should SSHT be viewed not only as a disorder of renal sodium handling, but also as a condition involving abnormal immune sodium sensing?
2. Does the SGK1/ENaC axis represent a central mechanism by which immune cells detect high-salt conditions and initiate inflammatory responses?
3. How does salt-driven immune activation contribute to renal inflammation and kidney injury?
4. Could targeting the SGK1/ENaC pathway provide a promising strategy for reducing renal injury in SSHT?
5. What are the existing challenges and limitations in exploring the SGK1/ENaC axis signaling pathway and its application in renal injury during SSHT?

In this article, we revisit SSHT as an immune sodium-sensing disorder and argue that the SGK1/ENaC axis may serve as a key mechanistic bridge between salt exposure, inflammation, and renal injury. Focusing on this pathway can help expound how immune activation interacts with kidney injury and provide new perspectives for its treatment.

## 2 Salt-sensitive hypertension as an immune sodium sensing disorder

SSHT has traditionally been explained by abnormal renal sodium handling and impaired vascular adaptation to salt loading (14, 15). These mechanisms remain important, but they do not fully explain why a high-salt environment is often accompanied by persistent inflammation and progressive target-organ injury (11, 16). Recent evidence supports a view that SSHT is not only a hemodynamic disorder, but also a disease in which immune cells directly respond to excessive sodium and promote disease progression (17, 18). In conclusion, sodium should be regarded not only as an electrolyte that affects volume balance, but also as a biologically active signal that can shape immune behavior (11, 19).

With the progress of research, new discoveries have gradually emerged, indicating that sodium can accumulate in interstitial tissues and directly activate APCs (20, 21). In dendritic cells, sodium enters through amiloride-sensitive channels and triggers a cascade that includes activation of NADPH oxidase, oxidative stress, and the formation of isolevuglandin-protein adducts (9, 10, 22). These modified proteins behave as neoantigens and promote cytokine release, creating conditions that enhance T-cell activation (22). This finding is important because it shows that immune activation in SSHT is not simply secondary to elevated blood pressure. On the contrary, immune cells can detect the sodium-rich environment itself and initiate inflammatory signaling (9, 17, 23).

Immune cells play a significant role in this process. High salt has been shown to favor pro-inflammatory T-cell development and promote tissue inflammation, especially those involving T helper 17-related responses, while SGK1 serves as an important salt-responsive signaling molecule in these cells (24). In mice models, deletion of SGK1 in T-cells blunts the BP response and markedly reduces renal and vascular inflammation as well as end-organ damage (25). These observations support the idea that immune sodium sensing is functionally relevant, not just a molecular curiosity. Once activated, immune cells can amplify inflammation through cytokine release and tissue infiltration, thereby linking salt exposure to kidney injury (25).

Taken together, these studies support a framework that SSHT is considered an immune sodium-sensing disorder. This viewpoint not only fails to replace classical kidney and vascular models, but also combines them with immune mechanisms (26). The kidney is therefore better understood as a site where sodium handling, inflammation, and tissue injury interact (26). This conceptual shift is important because it helps explain why high salt can produce renal damage out of proportion to BP alone and sets the stage for examining the SGK1/ENaC pathway as a central immune sodium sensing mechanism (27, 28).

## 3 The SGK1/ENaC axis in immune cells

**Figure 1:**
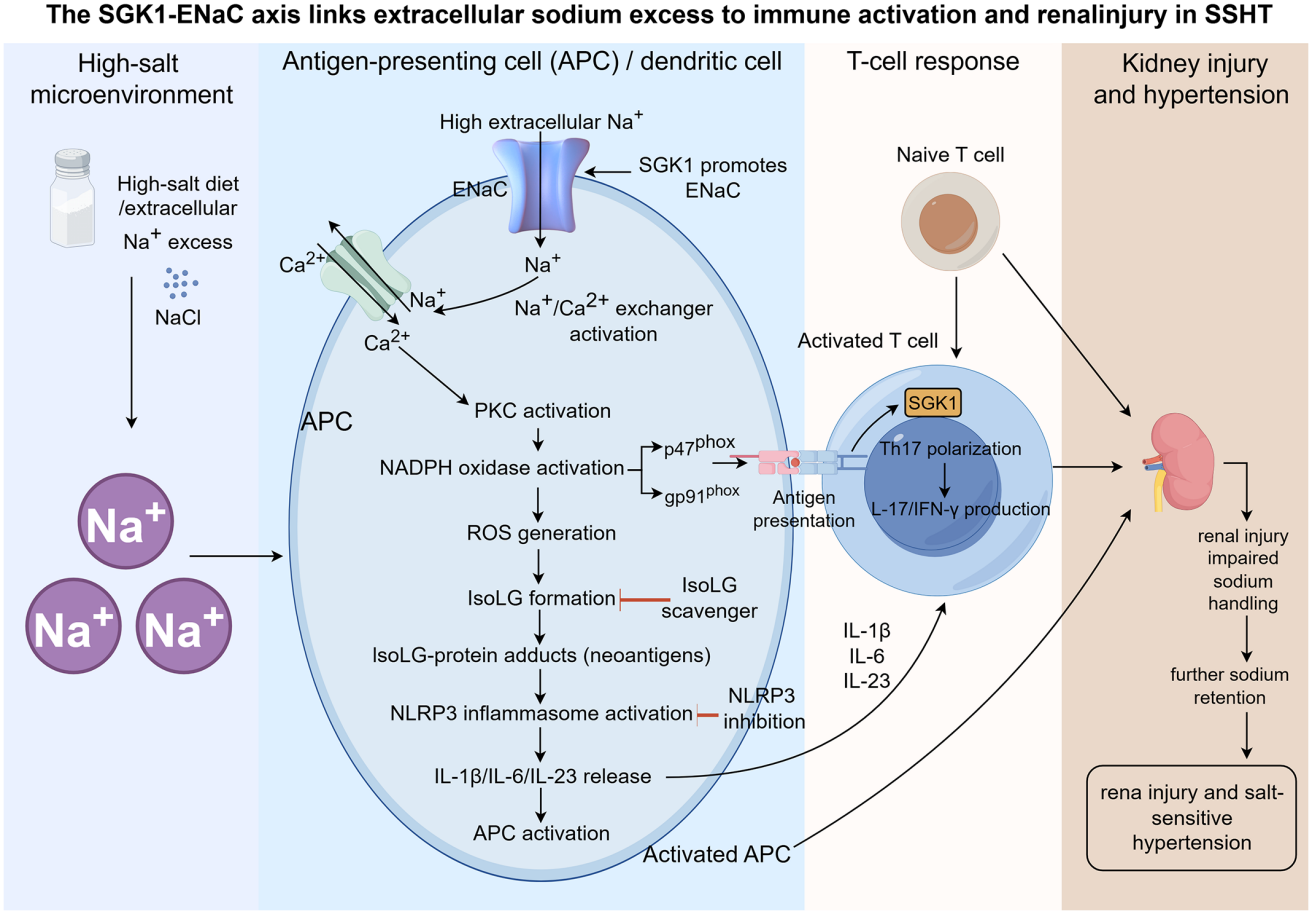
Delineates the SGK1–ENaC axis as a mechanistic bridge between interstitial sodium excess and hypertensive immune activation. High dietary salt intake elevates extracellular Na^+^, which enters antigen-presenting cells (APCs) via SGK1-promoted epithelial sodium channel (ENaC) (9, 10). This triggers Na^+^/Ca^2+^ exchanger activation, leading to increased intracellular Ca^2+^, protein kinase C (PKC) activation, and subsequent nicotinamide adenine dinucleotide phosphate (NADPH) oxidase activation (9, 29). Activated NADPH oxidase (via p47phox and gp91phox subunits) generates reactive oxygen species (ROS), which promotes isolevuglandin (IsoLG) formation and IsoLG-protein adduct (neoantigen) generation (29). These neoantigens activate the NLR family pyrin domain-containing 3 (NLRP3) inflammasome, driving release of interleukin (IL)-1β, IL-6, and IL-23, and promoting APC activation (9, 29). Activated APCs present antigens to naive T cells, and together with IL-1β, IL-6, and IL-23, drive Th17 polarization and production of IL-17/IFN-γ, a process mediated by SGK1 in T cells (9, 10). Activated T cells induce renal injury, impairing sodium handling and causing further sodium retention, which sustains renal injury and salt-sensitive hypertension (9, 10). The IsoLG scavenger and NLRP3 inhibition represent points of negative regulation in this cascade (29).

Among the pathways currently linked to immune sodium sensing, the SGK1–ENaC axis—predominantly restricted to antigen-presenting cells is one of the best defined (the specific mechanism can refer to **Figure 1**). Its strongest experimental support comes from APCs, especially dendritic cells and monocyte-derived myeloid cells (9, 10, 29). Under high-salt conditions, SGK1 expression increases in CD11c^+^ APCs and promotes the expression and assembly of ENaC-α and ENaC-γ, which increases sodium entry into these cells (9). In parallel, SGK1 also enhances activation of the NADPH oxidase pathway. Importantly, either genetic deletion of SGK1 in CD11c^+^ cells or pharmacologic SGK1 inhibition prevents salt-induced ENaC upregulation, reduces oxidative signaling, and protects mice from renal inflammation, endothelial dysfunction, and SSHT (9). These findings place SGK1 upstream of ENaC-dependent immune activation and suggest that this kinase-channel pair functions as a central sodium-responsive module in myeloid immune cells (9, 29).

Mechanistically, this pathway plays an important role in APCs. High extracellular sodium enters APCs through amiloride-sensitive ENaC, and this promotes intracellular signaling through the Na^+^/Ca^2+^ exchanger, leading to calcium influx (30). Amilolide is a non-selective sodium transport inhibitor that can block multiple pathways such as ENaC and NHE1 at high concentrations (31). Therefore, relying solely on Amilolide sensitivity as evidence for ENaC specificity has limitations. It should be noted that some *in vitro* studies in this field have used ultra-physiological high sodium conditions such as 190 mM, which are accompanied by significant high osmotic stress. The observed immune activation may be partially derived from osmotic pressure effects rather than specific sodium signals (32). Under salt sensitive hypertension in the body, the sodium concentration in the interstitial spaces and immune cells only increases by 10–20 mM compared to physiological levels. Within this concentration range, which is closer to pathological levels, SGK1/ENAC dependent sodium influx, oxidative stress, and inflammatory activation have been repeatedly confirmed. The rise in intracellular calcium activates protein kinase C (PKC), which then promotes assembly and activation of the NADPH oxidase complex through phosphorylation of p47^phox^ and its interaction with gp91^phox^ (30). The resulting oxidative burst generates reactive oxygen species and highly reactive lipid aldehydes known as isolevuglandins (IsoLGs) (10, 33). IsoLGs rapidly adduct to lysine residues on cellular proteins, producing modified proteins that behave as neoantigens (9, 34). APCs exposed to excess sodium then show higher levels of IL-1β, IL-6, and IL-23, and they acquire a greater capacity to activate T cells (34, 35). In this way, the SGK1/ENaC axis not only allows sodium entry, but also converts a salt signal into a durable inflammatory program.

More recent studies link ENaC-dependent sodium entry to NLRP3 inflammasome activation (10, 36). In human monocytes, high sodium increases transcription of NLRP3-related genes, pyroptotic and apoptotic caspases, and IL-1β (21, 36). In mice models, this inflammasome response is ENaC- and IsoLG-dependent, and NLRP3-deficient mice develop a blunted hypertensive response to salt exposure. Notably, the phenotype can be restored by adoptive transfer of NLRP3-intact APCs (9, 36). Human translational studies further support the relevance of this pathway (36). In patients with salt-sensitive hypertension, the proportion of IsoLG-positive APCs changes with salt loading and depletion, and human APCs express ENaC subunits together with SGK1 (36). Taken together, these findings argue that the SGK1–ENaC axis is not merely an *in vitro* observation, but a biologically meaningful pathway that tracks with salt sensitivity in humans.

The sodium-sensing mechanism in T-cells differs from that in APCs. SGK1 clearly contributes to salt-driven T-cell pathogenicity. Research show that modest increases in sodium can induce SGK1, increase IL-23 receptor expression, and promote Th17 differentiation, while deletion of SGK1 in T-cells blunts BP elevation and markedly reduces renal and vascular inflammation in experimental hypertension (25, 37, 38). However, current evidence suggests that the upstream sodium transporter in T cells is more likely to involve Na^+^-K^+^-2Cl- cotransporter 1 (NKCC1) than ENaC (9, 25). For that reason, the SGK1–ENaC axis is an APC-specific mechanism and should not be generalized to all immune cells. Based on the available data, it is best viewed as an APC-selective core pathway of immune sodium sensing that then amplifies downstream T-cell responses, rather than as a universal mechanism shared identically by all immune cell subsets (9, 39, 40). This interpretation is both more accurate and more useful, because it explains how local sodium excess can be translated into APC activation, T-cell polarization, and finally renal inflammation and injury in SSHT. In addition, SGK1 not only acts on immune cells, but also plays a core regulatory role in sodium transport in renal tubular epithelium. SGK1 can upregulate the expression of renal tubular ENaC membrane by inhibiting NEDD4-2-mediated ubiquitination degradation, while activating the WNK-OSR1/SPAK signaling axis, enhancing the expression and activity of proximal tubular NHE3 and distal tubular NCC, directly promoting sodium reabsorption and reducing sodium excretion (41–43). This effect enables the activation of SGK1 signals within immune cells to be transmitted across cells to the renal tubules, directly connecting immune sodium perception with abnormal renal sodium processing, forming a key molecular node for immune renal tubular interaction.

## 4 From immune activation to renal injury in salt-sensitive hypertension

Once immune responses are engaged, renal injury in SSHT should not be regarded as a passive consequence of pressure overload alone. Instead, the kidney becomes an active site where inflammatory cells, cytokines, oxidative stress, and altered tubular transport reinforce one another and progressively impair sodium homeostasis (11, 27). Regal et al. found that in Dahl salt-sensitive rats have shown that a high-salt diet is accompanied by marked accumulation of T-cells and mononuclear cells within the renal cortex and medulla, together with albuminuria, glomerular injury, tubular casts, and worsening hypertension (44). Importantly, suppression of lymphocyte infiltration attenuates both renal damage and BP elevation, indicating that infiltrating immune cells are not merely secondary markers of injury but functional drivers of disease progression (45).

Renal pro-inflammatory microenvironment is the core pathological feature in hypertensive kidney injury (46, 47). Activated T-cells and myeloid cells release mediators such as IL-17A, IFN-γ, TNF-α, and IL-6, which amplify local tissue stress and sustain leukocyte recruitment (46, 47). In the Dahl salt-sensitive model, renal IL-6 is markedly increased during salt loading, and IL-6 neutralization reduces macrophage and monocyte accumulation while also lessening albuminuria, glomerular injury, tubular damage, and the hypertensive response (48). These findings suggest that cytokine signaling does not simply accompany renal injury, but actively shapes the composition and persistence of the inflammatory infiltrate. More broadly, immune cells in the hypertensive kidney promote oxidative stress and endothelial dysfunction, thereby worsening regional hypoxia and making the renal parenchyma more susceptible to structural injury (46, 49).

The link between inflammation and disordered sodium handling is especially important in salt-sensitive disease. Immune-derived cytokines can directly alter tubular transporter activity, thereby converting inflammation into sodium retention (50). IL-17A has been shown to increase the abundance or activation of major renal sodium transport pathways, including sodium hydrogen exchanger 3 (NHE3) in the proximal tubule and sodium–chloride cotransporter (NCC) in the distal nephron, with downstream effects that favor sodium reabsorption and impair natriuresis (50). Norlander et al. Found that mice lack IL-17A have reduced BP elevation, retained sodium response, and decreased NHE3 expression (50). Likewise, studies examining IFN-γ and IL-17A together have shown that these cytokines are required for full activation of renal sodium transporters during hypertensive stress (30). Their absence blunts the expected enhancement of tubular sodium reabsorption, providing a mechanistic explanation for how adaptive immune activation can sustain salt-sensitive BP elevation (50, 51).

These tubular effects have important consequences for renal structure. When inflammatory signaling promotes inappropriate sodium retention, the kidney is exposed not only to persistent hemodynamic stress but also to ongoing metabolic and oxidative burden (23, 52). This contributes to endothelial injury, interstitial inflammation, tubular epithelial stress, and progressive fibrotic remodeling (53). In this setting, the kidney gradually loses its ability to excrete sodium efficiently, so that salt sensitivity becomes further amplified (54). This creates a feed-forward loop in which immune activation accelerates renal damage, damaged renal tissue becomes less capable of pressure natriuresis, and impaired sodium excretion then favors additional hypertension and inflammatory cell recruitment (55–57). Reviews of immune actions in the hypertensive kidney consistently support this concept that renal inflammation is functionally linked to sodium retention rather than being an unrelated by-product of elevated BP (23, 55).

Taken together, current evidence supports a model in which the transition from immune activation to renal injury is mediated by two interrelated processes: I Sustained establishment of a pro-inflammatory microenvironment in the kidney; II Dysregulated cytokine signaling that modulates renal tubular sodium transport. Experimentally validated links include: high salt activates the APC–SGK1/ENaC axis to trigger inflammatory responses (9, 22, 36); IL-17A, IFN-γ and other mediators upregulate NHE3 and NCC to enhance sodium reabsorption (50, 51); sodium retention further exacerbates hypertension and renal injury (44, 47). Mechanisms that remain to be fully clarified include: direct molecular crosstalk by which renal injury reciprocally promotes immune cell infiltration; the spatiotemporal causal relationship between local renal sodium accumulation and inflammatory activation; and the quantitative contribution of SGK1 to immune–tubular crosstalk. This feedback loop is biologically plausible but requires further experimental evidence to establish a complete causal cascade.

## 5 Challenges and limitations

Despite the growing evidence implicating immune sodium sensing in SSHT, several limitations should be considered before the SGK1/ENaC axis is viewed as a comprehensive explanation for renal injury. First, salt sensitivity itself remains difficult to define consistently in clinical research. Available protocols differ in sodium loading, depletion, BP thresholds, and observation periods, and no routine clinical method currently provides sufficient sensitivity and specificity for identifying salt-sensitive individuals (58, 59). This heterogeneity complicates comparisons across studies and weakens efforts to align mechanistic findings with patient phenotypes.

Second, much of the strongest evidence still comes from mice models rather than from large human cohorts. Beusecum et al. found that mice lacking SGK1 in CD11c^+^ cells are immune to nephritis and endothelial dysfunction (9). Although there have been numerous animal studies, clinical research is still insufficient (9, 36). Therefore, the specific manifestations of these immune features at different stages of gender, race, comorbidity burden, and kidney disease are still uncertain.

Third, the biological specificity of this SGK1/ENaC remains incomplete. The SGK1/ENaC model is most strongly supported in APCs, but it should not yet be assumed to function identically across all immune cell subsets (9, 25, 29). In addition, ENaC composition differs between species; for example the δ subunit appears relevant in human APCs, yet it is not functional in standard mouse models (60, 61). This limits direct translation from mouse experiments to human disease. Moreover, SGK1 and ENaC are not immune-restricted targets. Both also participate in renal epithelial sodium handling and vascular regulation, making it difficult to isolate immune-specific effects *in vivo* (62, 63).

Finally, the relationship between tissue sodium accumulation and inflammation is still not fully resolved (64, 65). Although 23-Sodium magnetic resonance imaging (^23^Na-MRI) has improved noninvasive assessment of tissue sodium, accurate quantification still depends on specialized hardware, acquisition strategies, and post-processing, and kidney-specific applications remain technically challenging (66, 67). These limitations make it difficult to determine whether local sodium accumulation is a primary driver of renal inflammation or, in some contexts, a consequence of impaired renal function (65). Therapeutic translation is therefore still premature, especially because systemic ENaC inhibition carries a recognized risk of hyperkalemia (64, 66).

## 6 Future directions

The current research on salt sensitivity has limitations in diagnosis and mechanistic understanding, and further research is crucial to promote the development of the field. First, we need better ways to identify who is truly salt-sensitive (22, 68). At present, salt sensitivity is hard to diagnose in routine medical treatment, because current testing is time-consuming and not standardized (69). Future studies should combine blood BP, clinical features, and simple biomarkers. Recent human work suggests that circulating IsoLG-positive APCs and related eicosanoid signals may help identify salt-sensitive patients without a full salt-loading protocol (36). Genetic markers may also be useful in selected groups, but they still need broader validation (70).

Second, more evidence is needed directly from human tissues. Most mechanistic work still comes from animal models, while human data are limited. Future studies should test whether the same immune pathways are active in blood, urine, and kidney tissue from patients with SSHT, especially at different stages of kidney damage (11, 71). It will also be important to include women, different ancestry groups, and normotensive salt-sensitive individuals, because salt sensitivity is not confined to one clinical population (72).

Third, newer technologies may help us understand where injury starts and which cells matter most. Single-cell and spatial transcriptomic approaches are now able to map cell-specific and region-specific changes in the kidney during hypertensive injury (73, 74). These tools could show how immune cells, tubules, endothelium, and fibrotic cells interact over time, and may help define which parts of the kidney are most sensitive to sodium-driven inflammation (65, 75). In parallel, ^23^Na-MRI offers a noninvasive way to study tissue sodium storage in humans, although it is still mainly a research tool (65). Together, these approaches could link local sodium accumulation to local immune and renal injury more directly than before.

Finally, precise treatment strategies are also important. Broad ENaC inhibition can lower sodium reabsorption, but it may also cause side effects such as hyperkalemia (76). Future therapies should aim for cell-specific targeting of immune sodium-sensing pathways rather than general blockade of renal sodium transport. The long-term goal is not only to lower BP, but also to interrupt the cycle of sodium retention, inflammation, and kidney injury that drives disease progression (77).

## 7 Discussion

In summary, the SGK1/ENaC axis provides a useful framework for understanding how excess salt can be translated into immune activation and then into renal injury during SSHT. Current evidence supports a model in which sodium-sensitive immune pathways, especially in APCs, promote downstream T-cell activation, cytokine release, and progressive kidney damage rather than acting as isolated immune events. These inflammatory signals can also disturb renal sodium transport, helping to explain why kidney injury and salt retention often worsen together in a self-reinforcing cycle.

Collectively, this review integrates current evidence to propose a unified mechanistic framework for salt-sensitive hypertension that connects immune sodium sensing, renal sodium handling, and progressive renal injury. A proposed mechanistic cascade supported by existing evidence can be summarized in four interrelated steps. Excess dietary salt raises interstitial sodium concentration, which is detected not only by renal tubular epithelium but also by renal immune cells, especially antigen-presenting cells (22, 30, 36). The SGK1/ENaC axis in APCs acts as an important sodium-sensing module to trigger oxidative stress, IsoLG adduct formation, NLRP3 inflammasome activation, and pro-inflammatory cytokine release (9, 36). Inflammatory mediators including IL-17A, IFN-γ, and IL-6 act on renal tubules to upregulate NHE3, NCC, and ENaC, enhancing sodium reabsorption and impairing natriuresis (50, 51). Meanwhile, SGK1 directly promotes tubular sodium transporters, forming a key molecular link between immune activation and renal sodium retention (9, 24). Sodium retention, hypertension, renal inflammation, and tissue injury amplify each other, establishing a feed-forward cycle that drives persistent salt sensitivity and progressive renal damage (26, 55). This integrated model redefines SSHT as a crosstalk disorder between immune sodium sensing and renal tubular regulation, in which the SGK1/ENaC axis functions as a major mechanistic node. This framework unifies classical renal hemodynamic theories with modern immune mechanisms, providing a comprehensive interpretation for salt-sensitive hypertension and associated renal injury.

At the same time, this model should be viewed with appropriate caution. The strongest mechanistic data still come from preclinical studies, and the human evidence, although promising, remains limited. It is therefore too early to treat the SGK1/ENaC axis as the single explanation for all forms of SSHT. Still, this pathway highlights an important shift in thinking that SSHT is not only a disorder of renal sodium handling but also a disease shaped by immune dysfunction. This perspective may open new opportunities for patient stratification and more targeted therapies in the future.

## Data Availability

The original contributions presented in the study are included in the article/supplementary material. Further inquiries can be directed to the corresponding author.
